# Mechanical circulatory assist devices: a primer for critical care and emergency physicians

**DOI:** 10.1186/s13054-016-1328-z

**Published:** 2016-06-25

**Authors:** Ayan Sen, Joel S. Larson, Kianoush B. Kashani, Stacy L. Libricz, Bhavesh M. Patel, Pramod K. Guru, Cory M. Alwardt, Octavio Pajaro, J. Christopher Farmer

**Affiliations:** Department of Critical Care Medicine, Mayo Clinic Hospital, 5777 E. Mayo Blvd, Phoenix, AZ 85054 USA; Division of Cardiovascular and Thoracic Surgery, Mayo Clinic Hospital, Phoenix, Arizona USA; Division of Nephrology and Hypertension, Mayo Clinic, Rochester, Minnesota USA; Division of Pulmonary and Critical Care Medicine, Mayo Clinic, Rochester, Minnesota USA; Department of Critical Care Medicine, Mayo Clinic Jacksonville, Florida, USA

**Keywords:** Biventricular assist device, Cardiac arrest, Device failure, Gastrointestinal bleeding, Hemodynamic, Hypotension, Mechanical circulatory assist devices, Sepsis, Shortness of breath, Total artificial heart

## Abstract

Mechanical circulatory assist devices are now commonly used in the treatment of severe heart failure as bridges to cardiac transplant, as destination therapy for patients who are not transplant candidates, and as bridges to recovery and “decision-making”. These devices, which can be used to support the left or right ventricles or both, restore circulation to the tissues, thereby improving organ function. Left ventricular assist devices (LVADs) are the most common support devices. To care for patients with these devices, health care providers in emergency departments (EDs) and intensive care units (ICUs) need to understand the physiology of the devices, the vocabulary of mechanical support, the types of complications patients may have, diagnostic techniques, and decision-making regarding treatment. Patients with LVADs who come to the ED or are admitted to the ICU usually have nonspecific clinical symptoms, most commonly shortness of breath, hypotension, anemia, chest pain, syncope, hemoptysis, gastrointestinal bleeding, jaundice, fever, oliguria and hematuria, altered mental status, headache, seizure, and back pain. Other patients are seen for cardiac arrest, psychiatric issues, sequelae of noncardiac surgery, and trauma. Although most patients have LVADs, some may have biventricular support devices or total artificial hearts. Involving a team of cardiac surgeons, perfusion experts, and heart-failure physicians, as well as ED and ICU physicians and nurses, is critical for managing treatment for these patients and for successful outcomes. This review is designed for critical care providers who may be the first to see these patients in the ED or ICU.

## Background

Mechanical circulatory assist devices are now commonly used to support the failing heart: as a bridge to transplant (BTT) to support cardiac function before heart transplantation; as a bridge to recovery to give the native heart a chance to recover; as a bridge to decision until a determination can be made regarding a patient’s eligibility for cardiac transplantation; as a bridge until a more definitive device can be implanted (e.g., a biventricular assist device [BiVAD] to a left ventricular assist device [LVAD]); and as destination therapy (DT) to support cardiac function for the remainder of a patient’s life [1, 2].

**Fig. 1:**
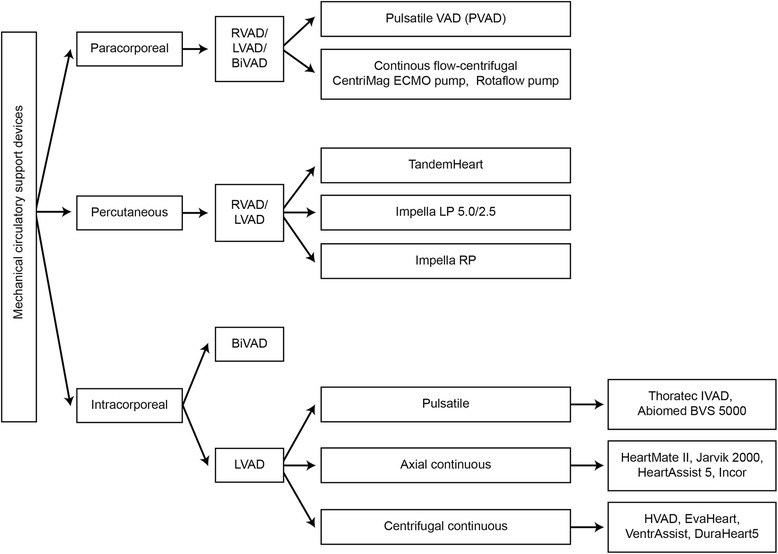
Classification of mechanical circulatory support devices. *BiVAD* biventricular assist device, *ECMO* extracorporeal membrane oxygenation, *LVAD* left ventricular assist device, *PVAD* pulsatile ventricular assist device, *RVAD* right ventricular assist device, *VAD* ventricular assist device. *BVS 5000* (Abiomed Inc.), *CentriMag* (Thoratec Corp.), *EvaHeart* (Evaheart, Inc. [available in Japan]), *HeartAssist5* (ReliantHeart [CE mark in Europe, FDA approval for humanitarian device exception in the United States for pediatric patients]), *HeartMate II* (Thoratec Corp.), *HeartWare HVAD* (HeartWare Inc.), *Impella* (Abiomed Inc.), *Incor* (Berlin Heart AG [available in Europe]), *Jarvik 2000* (Jarvik Heart, Inc.), *Rotaflow* (Maquet Holding BV & Co.), *TandemHeart* (CardiacAssist, Inc), *VentriAssist* (Ventracor, Ltd [available in Australia])

Mechanical circulatory assist devices restore tissue circulation by increasing blood supply, thereby improving organ function. However, they can be challenging to manage and are associated with complications, some of which are life-threatening. With increasing numbers of devices being implanted both for BTT and for DT, critical care and emergency physicians should become familiar with the different types of devices (Fig. 1) [3, 4], understand the unique physiology associated with them, be familiar with the clinical presentation of patients who experience complications, and know how to manage symptoms and complications.

## Device descriptions

**Fig. 2:**
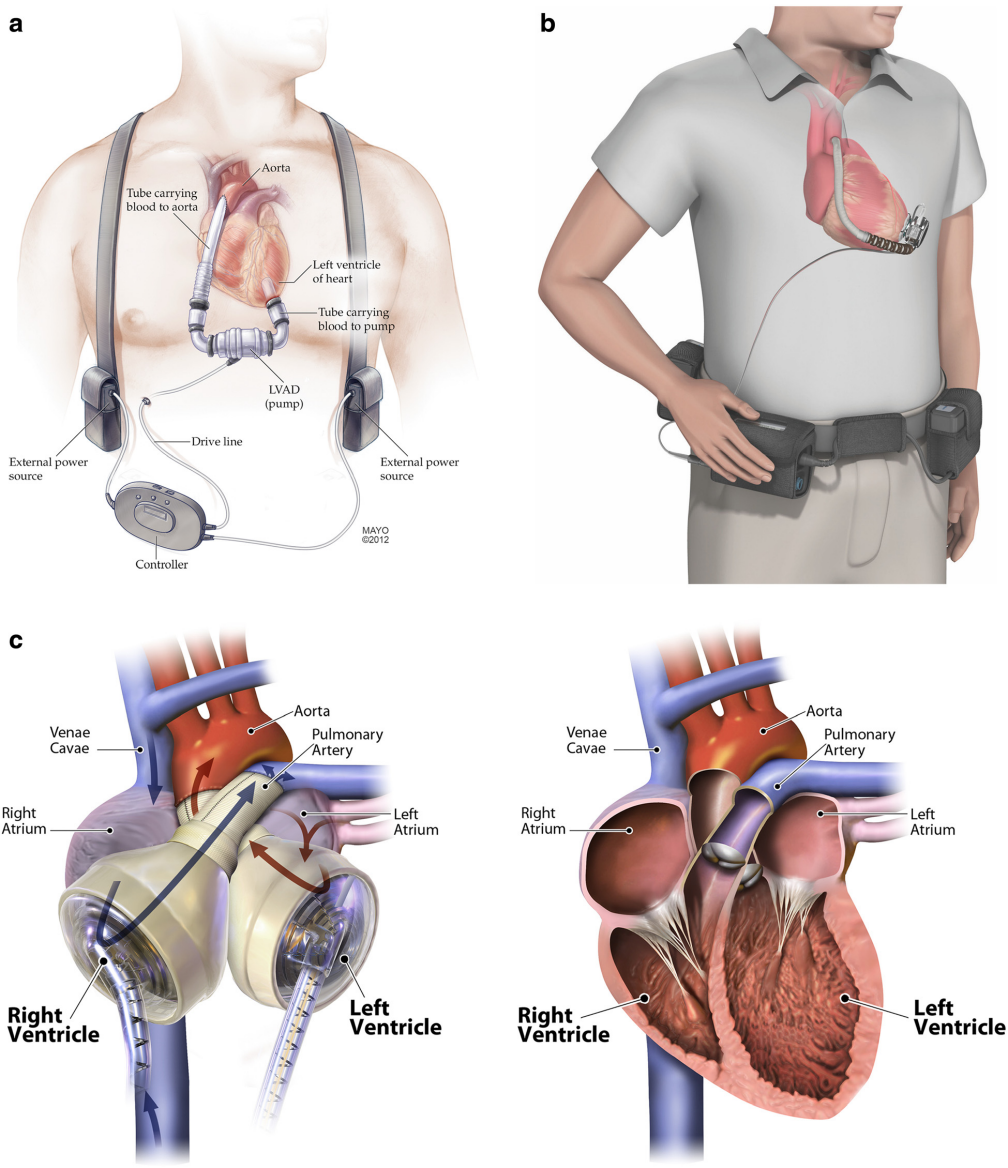
Most commonly used mechanical circulatory support devices. **a** The HeartMate II LVAD (Thoratec Corp.). **b** The HeartWare LVAD (HeartWare Inc.). **c** The SynCardia TAH (*left*) shown for comparison with the human heart (*right*) (SynCardia Systems, Inc.). *LVAD* left ventricular assist device, *TAH* total artificial heart. **a** From Mayo Foundation for Medical Education and Research; used with permission. **b** Provided by HeartWare; used with permission. **c** Image courtesy of syncardia.com; used with permission

Depending upon the indication, one of the following types of devices, often called *pumps*, may be implanted: paracorporeal or extracorporeal devices, which are placed outside the patient’s body, and intracorporeal devices, which are implanted in a preperitoneal position either above (in the pericardial space) or below the diaphragm (Fig. 2). All of the currently available implantable pumps have external controllers and power sources. Of the implantable devices, LVADs, as the name suggests, support left ventricular (LV) function in a situation where right ventricular (RV) function is adequate and RV assist devices (RVADs) support right ventricular function when LV function is adequate. For patients who require both RV and LV support, a BiVAD configuration can be used. BiVADs are composed of two separate devices: one for support of the RV and one for support of the LV. For patients with little remaining native cardiac function and no hope for a recovery, a total artificial heart (TAH) may be the only option. TAHs replace all functions of the native heart.

## Blood-flow characteristics

Mechanical circulatory assist devices produce either pulsatile or continuous blood flow. The first LVADs were pulsatile or displacement (pusher-plate) pumps. These devices were too large for an average-sized person, had a number of parts that could fail (e.g., valves, inflow and outflow conduits), and were associated with a variety of complications. They have been replaced with a new generation of continuous-flow pumps, which have inlet and outlet ports and a single, rotating element that imparts energy to the blood to increase arterial blood flow and pressure [5]. Blood is pulled into the impeller of the pump via an inflow cannula connected to the LV apex and delivered to the systemic circulation via an outflow cannula connected to either the ascending or descending aorta. Because the continuous-flow pumps have few moving parts, when properly functioning they seldom fail and are also associated with fewer complications than their predecessors, which has improved the quality of life for BTT and DT patients [6–8].

**Fig. 3:**
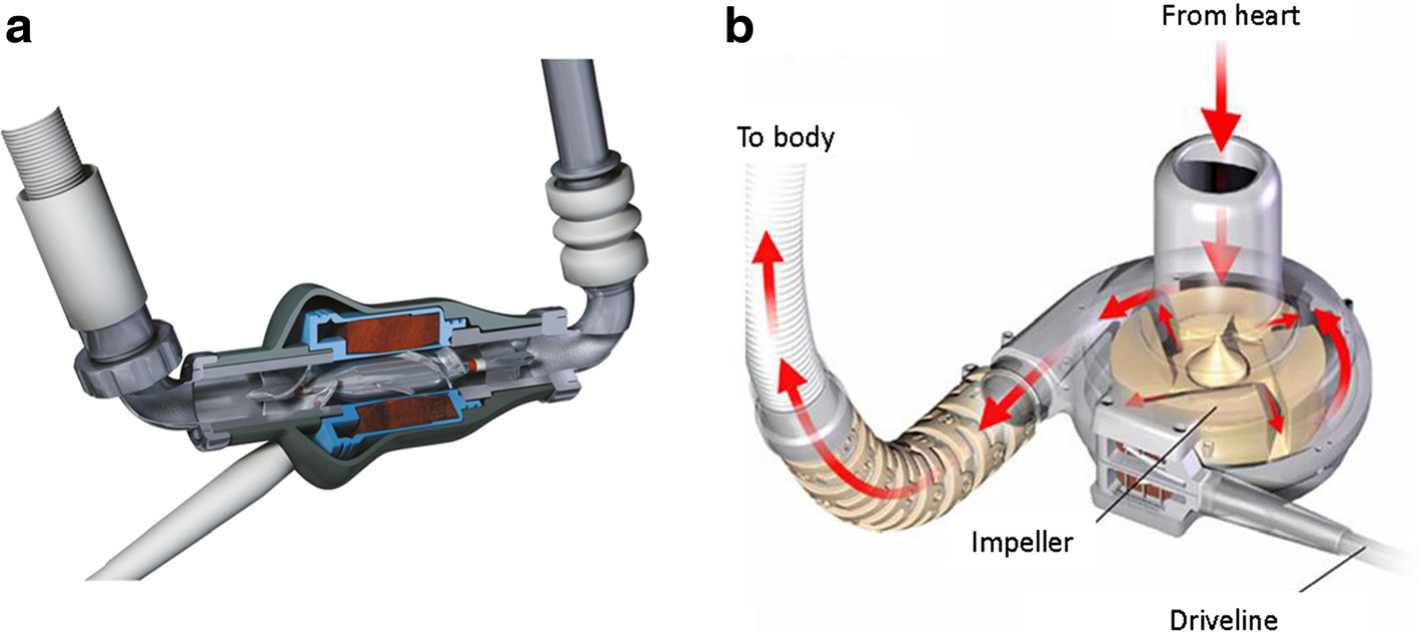
**a** Axial blood flow, shown in the HeartMate II LVAD (Thoratec Corp.). **b** Centrifugal blood flow, shown in the HeartWare LVAD (HeartWare Inc.). **a** Reprinted with the permission of Thoratech Corp. **b** Provided by HeartWare; used with permission

Continuous-flow LVADs available today produce two types of blood flow: centrifugal or axial [9]. In a centrifugal pump, blood is captured between rotating blades, which spin and, basically, throw the blood tangentially out from the blade tips (an induced force). In an axial-flow device, the rotating impeller operates like a propeller in a pipe. This mechanism can be thought of as an auger, trying to screw itself into the blood coming through the inlet cannula against the resistance force at the outlet cannula to overcome the difference between preload and afterload (Fig. 3). The two most commonly used devices today are the HeartMate II (Thoratec Corp.), an axial-flow device, and the HeartWare HVAD System (HeartWare Inc.), a centrifugal-flow device.

## Definitions

The following selected definitions are important to the understanding of how mechanical support devices operate.

### RPM

The revolutions per minute (RPMs), which determine pump flow, are set for each device by the heart failure physician caring for the patient. They are modified as flow needs change.

### Flow

The continuous flow from the LVAD is created by a spinning impeller, which generates forward flow [10]. A change in pump function or patient condition leads to changes in flow. The device flow is directly proportional to the rotor speed and inversely related to the difference of pressure in the inflow and outflow cannulas, i.e., Device flow = Rotor speed/(Pump_inflow_ − Pump_outflow_). Therefore, in addition to low RPM rate, a low flow rate could be caused by a number of conditions that would decrease preload to the device, such as decreased intravascular volume, RV failure, tamponade, thrombus, or kinking in the inflow cannula. In addition, low flow can occur with high afterload, such as when a patient has a hypertensive emergency or when the outflow cannula is obstructed.

### Pump power

LVAD pump power is a measure of the current and voltage applied to the motor and varies directly with pump speed and flow [10]. When flow is obstructed unrelated to contact with the rotor, power is reduced, whereas power is increased (and flows decreased) when thrombus forms on the rotor.

### Pulsatility index

The pulsatility index (PI) corresponds to the magnitude of flow pulse through the pump. The magnitude of flow pulse is measured and averaged over a 15-s interval to produce the PI for the HeartMate II (but not the HeartWare) [11]. The PI fluctuates with changes in volume status and the heart’s contractility. It increases when preload and contractility increase and decreases when blood volume and afterload are reduced and when there is an obstruction to inflow or outflow that causes low flow and abnormal power.

### Suction events

A suction event occurs when there is reduced filling of the pump (reduced preload), which increases negative pressure within the LV. When this occurs, part of the LV wall is *sucked over* and covers the pump’s inlet cannula; the pump then sounds an alarm and the speed will decrease to release the suction. Suction events are caused by low volume; RV failure or tamponade, which causes low LV filling; and inflow cannula obstruction. Suction events can lead to low LVAD flows and can trigger ventricular arrhythmias. The management includes decreasing the RPM rate and administering fluid [10].

Abnormal LVAD parameters and their diagnosis and management are listed in Table 1.

**Table 1:** Diagnosis and management of abnormal LVAD parameters

Abnormality	Cause	Interventions
High power	Pump thrombus	Anticoagulation therapy, pump exchange
Low power	Device problem	Check batteries, power source
High pulsatility index	Recovery of LV function	Wean LVAD support
	Lead damage	Check LVAD and driveline
Low pulsatility index	Worsening native ventricular function	Increase pump speed, inotropic therapy
	Hypovolemia	Administer fluid therapy
	Excess pump speed	Lower pump speed
High flow rate	Vasodilation (SALAD [sepsis/anaphylaxis/liver dysfunction/adrenal insufficiency/medications])	Identify and treat causes of sepsis; administer vasopressors for low mean arterial pressure
Low flow rate	Hypovolemia/bleeding RV failure/tamponade/hypertensive emergency	Administer intravenous fluids/blood
		Assess and treat
	Arrhythmias	Assess and treat
Suction events	All causes of low flow	Administer fluid therapy
	Excessive LV unloading	Lower pump speed

*LV* left ventricular, *LVAD* left ventricular assist device, *RV* right ventricular. Adapted from Feldman et al. [8] with permission

### Modes

Two modes can be set for the HeartMate II: fixed and auto. In fixed-rate mode, the pump rate is set close to the patient’s baseline value and does not vary, although the rate can be adjusted by using the system monitor. In auto mode, the LVAD responds to the patient’s activity and volume status, filling and emptying as needed to meet physiologic demand.

## Clinical presentations of patients with LVADs

Patients with LVADs who come to the emergency department (ED) or are admitted to the intensive care unit (ICU) usually have symptoms but not a clinical diagnosis. The following review will hopefully direct the provider in an ED or critical care unit to an approach for a differential diagnosis of symptoms for ventricular assist device (VAD)-specific and non-VAD-specific causes. Although this primer is meant for providers who see patients in an emergency setting, the approaches are also relevant for diagnosing and treating patients with new LVADs in a postoperative setting.

The common symptoms of patients with LVADs are nonspecific and include shortness of breath, hypotension, anemia, chest pain, syncope, hemoptysis, nausea, vomiting, diarrhea, gastrointestinal (GI) bleeding, jaundice, fever, chills, oliguria and hematuria, altered mental status, headache, seizure, and back pain. Other patients are seen for cardiac arrest, psychiatric issues, sequelae of noncardiac surgery, issues related to pregnancy, and trauma. The critical care physician may also need to evaluate patients for brain death and organ donation.

## Symptom approach

The following section describes a symptom-oriented approach for critical care providers to use in determining differential diagnoses and treating patients with LVADs.

### Shortness of breath

Shortness of breath (dyspnea or tachypnea) in an LVAD patient can be caused by the following three pathophysiologic conditions: reduced oxygen delivery, increased oxygen consumption, and inadequate carbon dioxide exchange. Causes of reduced oxygen delivery include hypoxic respiratory failure, acute anemia, and reduced cardiac output (CO). Increased oxygen consumption may be caused by adrenergic stress or vasodilation due to sepsis, anaphylaxis, acidosis, adrenal insufficiency, or liver dysfunction. Inadequate carbon dioxide exchange may be due to neurologic causes; neuromuscular issues; extrinsic causes, such as ascites; ileus; or reduced blood flow through pulmonary capillaries, such as in shock.

Patients who have shortness of breath should have their arterial blood gas and lactate levels measured, should be monitored with continuous pulse oximetry (although this may not be accurate because of continuous flow), and have a chest radiograph and, if necessary, a computed tomography (CT) scan. Supplemental oxygen (i.e., nasal cannula, face mask, high-flow oxygen, noninvasive positive-pressure ventilation, or mechanical ventilation) should be used according to the usual standard of care. When acute hypoxic respiratory failure is the cause of shortness of breath, it may be helpful to assess the effect of an increased fraction of inspired oxygen (Fio_2_), which will improve dead space versus increased positive end-expiratory pressure (PEEP), which will improve a pulmonary shunt. If neither increased Fio_2_ nor PEEP improves oxygenation, a cardiac shunt, such as an open patent foramen ovale, may be the cause and should be considered in the diagnosis of the shortness of breath.

The patient’s hematocrit and hemoglobin levels should be assessed to rule out bleeding and hemolysis as causes of shortness of breath. Patients with LVADs take anticoagulation medications and have a bleeding diathesis in addition to the risk of hemolysis from the device–blood interaction.

Reduced CO may also cause shortness of breath. Cardiogenic Shock needs to be considered in the differential diagnosis. These patients may also have co-existent hypotension (see the “Shock” section below for the approach to management of this condition).

The patient should be intubated for mechanical ventilation when hypoxia, hypercarbia, or acidosis worsens or when the airway cannot be protected. Rapid sequence intubation should be performed. Any anesthetic induction agent can be considered, although etomidate or a narcotic and benzodiazepine combination like fentanyl plus midazolam is often preferred. Lung-protective ventilation strategies which include a low tidal volume (Vt) and adequate PEEP should be used. A very high PEEP can cause worsening of RV dysfunction and should be avoided. Therapeutic management may include draining a pleural effusion, inserting a chest tube for pneumothorax, performing a bronchoscopy procedure, and optimizing hemodynamics.

### Shock

**Fig. 4:**
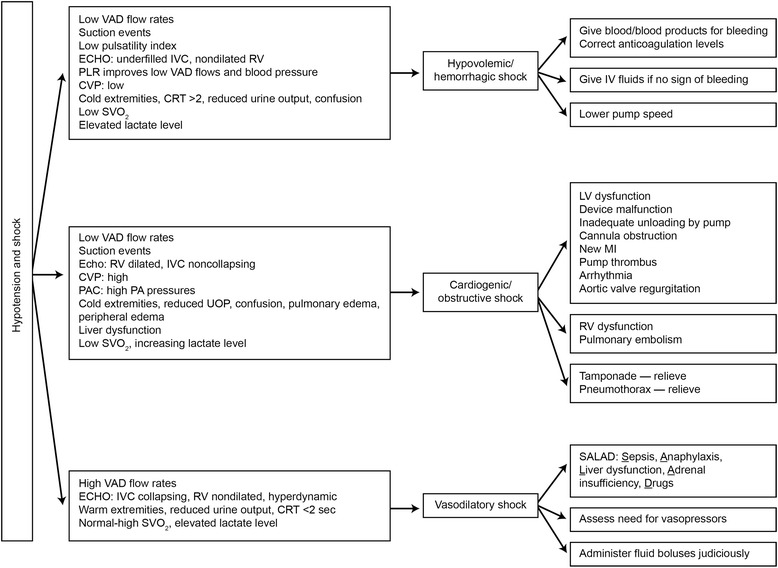
Differential diagnosis and management of hypotension and shock in patients with LVADs. *CRT* capillary refill time, *CVP* central venous pressure, *echo* echocardiography, *IV* intravenous, *IVC* inferior vena cava, *LV* left ventricular, *MI* myocardial infarction, *PA* pulmonary artery, *PAC* pulmonary artery catheter, *PLR* passive leg raising, *RV* right ventricle, *SVO*_*2*_ mixed venous oxygen saturation, *UOP* urinary output, *VAD* ventricular assist device

Shock (or hypotension) is defined as mean arterial pressure less than 60 mmHg, as measured by Doppler ultrasonography. Figure 4 summarizes making the differential diagnosis and the approach to treating LVAD patients who have hypotension. As in patients without LVADs, hypotension can be due to hypovolemia (often caused by hemorrhage), cardiogenic shock (often obstructive shock), or systemic vasodilation and the clinical examination may help determine the cause. Assessing the extremities (i.e., warm or cold) is critical to the diagnosis. Cold and mottled extremities indicate cardiogenic (obstructive) or hypovolemic (hemorrhagic) shock and patients with these conditions may also have reduced urine output, altered mental status, tachycardia, and shortness of breath.

The LVAD flow rate must also be assessed. An arterial catheter should be inserted for continuous mean arterial pressure (MAP) monitoring and is best placed via ultrasonographic guidance (continuous flow makes blind placement difficult). In addition, a central venous catheter may be needed to assess central venous pressure, preload, and preload responsiveness.

Bedside cardiac ultrasonography is very helpful for assessing hemodynamic parameters in a patient with an LVAD, especially when echocardiography has not been completed; it can be used for the following:

- Inferior vena cava and collapsibility
- RV size and function
- Position of the ventricular septum (flat and neutral)
- LV size and function
- Signs of pericardial effusion or tamponade
- Inflow cannula (if visualized) aligned with the mitral valve
- Outflow cannula (if visualized)
- Competency of the aortic valve (it should be competent and open intermittently, every second or third beat).

#### Cardiogenic and obstructive shock

Patients with LVADs can have cardiogenic or obstructive shock with acute heart failure and hemodynamic instability because of device malfunction or new cardiovascular dysfunction that is refractory to supportive therapy. These patients may require temporary circulatory support, either with an intraaortic balloon pump, a percutaneous LVAD (i.e., TandemHeart [CardiacAssist, Inc.]), or venoarterial extracorporeal membrane oxygenation.

The causes of cardiogenic shock and reduced CO, which cause hypotension in patients with LVADs, follow.

#### New-onset LV dysfunction

New-onset LV dysfunction may manifest as fluid overload or other signs of cardiogenic shock, such as cold, clammy extremities, altered mental status, shortness of breath, and reduced urine output. The cause of the dysfunction may be a new myocardial infarction (MI), arrhythmias, aortic valve degeneration causing aortic regurgitation, pericardial tamponade, or decreased LV function because of worsening contractility and fluid overload, or both. Management includes adjusting the LVAD speed, use of inotropic, diuretic, and antiarrhythmic agents, relieving tamponade, if present, and consideration of percutaneous LV support.

Patients with LVADs can have an acute MI from rupture of a coronary plaque or from paradoxical thromboembolism from a deep venous thrombosis, thromboembolism from an intracavitary thrombus in the LV or from the aortic root, and device failure, which leads to elevated LV filling pressures and impaired myocardial perfusion [12]. However, LV catheterization should be performed only after carefully weighing the risks and potential benefits to the patient. The potential benefits of a coronary intervention include (1) relieving symptoms, (2) preventing and treating of recurrent arrhythmias, (3) reducing ongoing myocardial damage, (4) supporting RV function if RV failure is present, and (5) avoiding LVAD alarms [13–15]. The potential disadvantages of cardiac catheterization are aortic valve or root thrombosis and wire entrapment in the inflow or outflow pump conduits or rotor [12].

#### RV failure

RV failure can occur in 5 to 10 % of patients after an LVAD implantation [16]. Echocardiographic parameters suggestive of RV failure include RV dilatation, reduced RV ejection fraction, reduced excursion of the tricuspid annulus as assessed on tissue Doppler imaging, and tricuspid regurgitation [12, 17]. Hemodynamic variables consistent with RV failure for a patient supported by an LVAD include a central venous pressure of more than 15 mmHg. If a pulmonary artery catheter is placed, it may show an elevated, mean pulmonary artery pressure greater than 25 mmHg and a high central venous pressure greater than 15 mmHg, with normal-to-low pulmonary capillary wedge pressure. Therefore, it is important to maintain RV function and reduce preload and afterload.

Several approaches are used to help maintain RV function. A low dose of an inotropic agent, such as dobutamine, epinephrine, or milrinone, is routinely administered and, when necessary, a vasoconstrictor agent is also administered (both support MAP). The ventricular septum should be maintained in a neutral position to avoid leftward or rightward shift, which can lead to a suction event. Maintaining the septum in its normal position can be done by carefully monitoring fluid volume, inotropes, and device settings and can be assessed by echocardiography. Too high a flow will shift the septum leftward and impair RV function; too low a flow will shift the septum rightward and cause increased left atrial pressure, which impairs RV function [18]. Anything that might cause pulmonary vascular resistance to increase, such as hypercarbia and hypoxia or high ventilation pressures or levels of PEEP, also needs to be controlled. Impaired RV function and increased pulmonary vascular resistance can be treated by giving sildenafil (25–50 mg every 8 h) or inhaled nitric oxide (20–40 ppm). In addition, the MAP should be maintained at a level higher than 60–70 mmHg to help maintain RV perfusion pressure. Volume administration needs to be controlled to avoid increased right ventricular end- diastolic pressure.

A central venous pressure greater than 18 mmHg in association with a cardiac index less than 2.0 L/min/m^2^ despite optimal medical therapy indicates the need for RVAD support. Either the Impella RP (Abiomed) or the TandemHeart (CardiacAssist, Inc.) right ventricular support device can be used for short-term percutaneous mechanical support until recovery of myocardial function or as a bridge to an RVAD [19].

#### Cardiac tamponade and tension pneumothorax

Hypotension and low device flow rates (whether or not suction events exist) may indicate developing cardiac tamponade, which can occur late. Bleeding diathesis and anticoagulation therapy are risk factors for tamponade. An urgent bedside echocardiogram may show the cause of the tamponade; however, transesophageal echocardiography (TEE) may be necessary for the diagnosis (e.g., when the windows on surface echocardiography are poor and unrevealing) and concern for regional or global tamponade still exists. When the cause of tamponade is a pneumothorax, a chest tube needs to be inserted emergently after needle decompression if the patient has hypotension and shortness of breath.

#### Arrhythmias

Patients may have atrial or ventricular arrhythmias or atrial and ventricular arrhythmias. Atrial fibrillation and atrial flutter have been reported to be present in patients with LVADs and are associated with worse clinical outcomes even without an increased risk of bleeding and thromboembolism. Although rapid atrial arrhythmias can be tolerated initially, loss of atrioventricular synchrony results in reduced ventricular filling and decompensated RV failure [20].

Rate control and anticoagulation are the primary goals of therapy and restoring normal sinus rhythm is important. Antiarrhythmic therapies for long-term use include β-blockers, such as metoprolol or sotalol, amiodarone, and digoxin. Cardioversion may be beneficial if the arrhythmia is of new onset. Radiofrequency catheter ablation has been shown to be far superior to antiarrhythmic medications for typical atrial fibrillation and atrial flutter, with cure rates approaching 95 % and minimal risk of procedural complications [20]. The frequency of arrhythmias may be reduced by careful management of hemodynamic parameters, volume status, and electrolyte balance.

Ventricular arrhythmias have been reported to occur in 22 to 59 % of LVAD recipients [8, 21]. The presentation of ventricular arrhythmias in patients with LVADs varies. Patients supported by LVADs may tolerate ventricular arrhythmias with minimal symptoms and stable hemodynamic parameters because of the LVAD’s ability to maintain CO independent of heart rate and atrioventricular synchrony [22]. However, ventricular arrhythmias caused by impaired RV filling can lead to inadequate LVAD flows and RV failure, hemodynamic deterioration, and even cardiac arrest [21]. Most patients require an automated implantable cardioverter defibrillator (AICD) for this reason and AICD shocks or overdrive antitachycardia pacing can occur during episodes of ventricular arrhythmias. Devices should be interrogated when patients have palpitations, shortness of breath, or hypotension or when the AICD fires. Reprogramming of the AICD may be needed to avoid unnecessary or inappropriate shocks.

Uncontrollable ventricular arrhythmias can be an indication for temporary extracorporeal membrane oxygenation, a BiVAD, heart transplantation, or a TAH. Intravenous amiodarone and lidocaine can be administered for acute symptoms. β-Blockers should only be used if patients do not have overt cardiogenic shock or heart failure. Oral amiodarone is often prescribed to suppress arrhythmias for outpatients or for hospitalized patients after the acute symptoms have been controlled with intravenous antiarrhythmic agents. For patients who cannot tolerate amiodarone, sotalol can be an option. For patients taking any of these medications, potassium and magnesium levels should be closely monitored to ensure electrolyte balance and QT-prolonging medications should probably be avoided.

Management of hemodynamic indices may reduce the occurrence of arrhythmias. For example, suction events may trigger ventricular arrhythmias; therefore, the device speed should be set to avoid excessive ventricular unloading. This can be done by using echocardiography to assess LV end-diastolic diameter and the degree of aortic valve opening [23, 24]. Cardioversion and radiofrequency ablation therapy after electrographic mapping have also been described as therapeutic options for managing arrhythmias [21].

#### Aortic valve degeneration

When used for extended periods, continuous-flow LVADs can lead to aortic valve degeneration and aortic regurgitation, as the alteration in blood-flow mechanics results in increased load and mechanical stress on the valve. The aortic valve leaflets can fuse either at the commissures or at the midportion of the leaflet, creating an incompetent valve [25, 26]. Such degeneration increases the risk of thromboembolism, infection, congestive heart failure, and cardiogenic shock. Aortic valve incompetence has been reported in more than 90 % of patients undergoing long-term ventricular assist device (VAD) support, although the time course varies [18, 27]. Echocardiography should be used to assess the status of the aortic valve.

For patients with aortic valve incompetence, vasodilatory antihypertensive agents should be prescribed to decrease afterload. Before heart failure develops, valve replacement or repair should be considered. In addition, the device speed can be adjusted so that the aortic valve opens intermittently, which will also improve hemodynamic indices and delay aortic valve degeneration [28].

#### Device failure (HeartMate II)

Patients with an LVAD are dependent on the device for their heart function and device failure may lead to circulatory arrest if the reason for the failure is not addressed emergently. Emergency and critical care providers should be aware of the critical steps associated with management of device failure [29].

- Call the LVAD coordinator and coordinating center.
- Check the instruction booklet and color of the tag on the system, which indicates the device type.
- Assess for battery alarms, advisory alarms, or hazard alarms.

#### Battery alarm

In a low-battery state, the device defaults to a fixed-rate mode of 8000 RPM (HeartMate II). The system will return to the set speed once adequate power is restored. The simple fix is to replace the batteries or switch to an alternative power source.

#### Advisory alarms

The yellow *system driver* signals low cell voltage and beeps every 4 seconds. When this occurs, the cell battery should be replaced and a system controller self-test performed. The green advisory alarm signals a *power-cable disconnection*; the power symbol and battery power bars flash with beeps every second. Cable connections to the power source should be checked and power leads should be assessed for damage and replaced if necessary. The red *hazard* alarm can indicate low flow and incorrect operation, or both, which may suggest hypovolemia, bleeding, tamponade, RV failure, hypertension, or obstruction of the cannulas. In these cases, both the patient and the device should be assessed emergently. The hazard alarm can also indicate that the driveline is disconnected from the controller, so the connections need to be checked. If there is a steady tone and no symbol, there is no power to the device. All connections should be checked immediately, e.g., the system driver connections to the device and the system driver and the power connections to the power source. In these circumstances, patients may be at risk of cardiopulmonary arrest because of minimal reserve in the cardiovascular system.

#### Driveline damage

The driveline can fail in the course of normal wear. When the driveline fails, a surgical procedure will often be required to replace it. Damage to the driveline wires may occur inside the abdomen where the damage is not accessible to visual inspection. When damage occurs externally, the outer silicone sheath may mask the damage to the driveline wires. The most typical clinical findings associated with driveline failure are the *red heart* alarm and a drop in the pump speed below the auto-speed–low set limit. Radiography showing driveline kinking or fraying in patients with unexplained alterations in LVAD performance suggests driveline damage and requires surgical management [30]. In some cases, minimally invasive procedures to repair the driveline have been done with a special sleeve-expander tool and expandable latex tubing to stabilize the driveline, without requiring pump exchange [31]. For patients who present to the ED in circulatory arrest, individual stripping and reconnection of the color-coded driveline wires using hemostats, electric tape, and cardboard has been successful, with reinstitution of flow from the LVAD [32].

#### Cannula obstruction

Obstruction of a cannula can also cause low device flow, even with adequate volume. In some instances, the cannula is not positioned properly. For example, when the inflow of the cannula points toward the septum or LV free wall (rather than toward the mitral valve), it can become intermittently blocked by septal shift. When a cannula is obstructed, there will be reduced power consumption and the VAD may make a *chattering* noise as it tries to fill. When echocardiography is not sufficient to make the diagnosis, patients may need to undergo CT angiography, ventriculography (with intravascular ultrasound), or both in the cardiac catheterization laboratory. Often an obstructed cannula must be surgically repaired, although balloon angioplasty with stenting of the outflow graft is a possible nonsurgical option [33].

#### Device thrombosis

One of the common causes of a low CO state is device thrombosis, which occurs in approximately 8 % of implanted, continuous-flow LVADs [34]. Device thrombosis can obstruct inflow or outflow and can also interfere with the motor. The thrombosis can originate within the device but can also result from a thrombus elsewhere being pulled into the LVAD [35]. Device thrombosis can develop even when patients are fully anticoagulated and taking antiplatelet therapy because the LVAD causes a chronic hypercoagulable state. A low or therapeutic international normalized ratio alone is not a good indicator of device thrombus formation [36]. Predisposing factors include blood–device surface interactions, shear stress of blood flow because of altered blood-flow dynamics (continuous flow), cannula malposition, acquired von Willebrand factor deficiency, and heparin-induced thrombocytopenia [37].

Patients with a thrombosed device may have signs of cardiogenic shock, including shortness of breath, hypotension, and tachycardia. On auscultation, there may be scratchy, grating, and rough sounds that are caused by device thrombus. In addition, there can be power spikes and low-flow alarms from the device and an increased native PI from the aortic valve’s opening or a significantly increased pulse pressure. Results of laboratory tests that reveal evidence of hemolysis (lactase dehydrogenase [LDH] levels greater than three times the upper limit of normal, plasma free hemoglobin >40 mg/dL, or both) should raise concern for possible thrombus. A plain chest radiograph may show malposition of the inflow cannula, a misaligned outflow graft, or pulmonary vascular congestion suggestive of heart failure and echocardiography can document signs of suboptimal LV unloading, including a dilated ventricle, severe mitral regurgitation, and frequent aortic valve opening or elevated RV systolic pressure or both. Serial recording of LV end-diastolic diameter with increasing VAD speeds (ramp study) may diagnose device thrombus or other obstructions to blood flow within the rotary pump and cannula system [38]. A filling defect in the inflow or outflow cannulas can also be visualized on CT angiography [39].

Patients with an LVAD that has developed thrombosis will develop LV dysfunction and, ultimately, cardiogenic shock and will need increased anticoagulant medications, thrombolytic therapy, or a device exchange. To stabilize or lower the LDH, the target value for the international normalized ratio can be increased and aspirin (325 mg) or a second antiplatelet agent, or both, can be considered. Eptifibatide, a glycoprotein IIb/IIIa inhibitor, has been used to inhibit platelet aggregation but it has led to increased risk of bleeding [40]. Thrombolytic therapy has also been used successfully [15, 18, 41]. If other measures fail, device exchange or urgent transplantation may be considered [13]. The cornerstones of treatment for heparin-induced thrombocytopenia are discontinuation of heparin and use of alternative anticoagulant agents, such as factor Xa inhibitors (e.g., fondaparinux) and direct thrombin inhibitors (e.g., bivalirudin and argatroban) [42, 43].

### Anemia

Anemia is common in patients with LVADs and may be a result of an episode of acute bleeding or hemolysis or may be chronic and associated with heart failure or nutritional or iron deficiencies.

#### Bleeding

When bleeding is suspected, the source should be determined. A complete blood count, coagulation profile, and type and screen should be ordered. Any coagulopathy should be corrected and a blood transfusion may be required. Platelets should be given to patients taking aspirin and fresh frozen plasma and vitamin K should be given to patients taking warfarin. Desmopressin may be used if the patient is uremic. Angiographic approaches to stop bleeding, e.g., retroperitoneal or gastrointestinal, should be explored. Prothrombin complex concentrate and factor VII should be considered as a last resort and only if the patient is in hemorrhagic shock. This is particularly important because the risk of device thrombosis is very high when these agents are used.

#### Hemoptysis

A variety of conditions can cause hemoptysis, including RV failure, coagulopathy (anticoagulant-related), thrombocytopenia, liver dysfunction, disseminated intravascular coagulation, bronchitis, airway trauma, a foreign body, and infection. An arteriobronchial fistula of the outflow graft, which caused hemoptysis, has also been reported [44]. When patients have hemoptysis, other causes of acute bleeding (both external and internal) should be ruled out. If there are no signs of external bleeding, bleeding needs to be ruled out in the intracranial, intrathoracic, intraabdominal, and pelvic compartments, and muscle compartments of the extremities.

#### Hemolysis

In recent years, hemolysis has become an increasingly recognized complication of LVAD support, with a prevalence of 0.5 to 18 % [45]. Hemolysis after LVAD implantation is associated with an extremely high 1-year mortality that is more than two times greater than that observed for patients (HeartMate II) who do not have hemolysis [45]. Increased shear stress on red blood cells as they pass through the device is the main mechanism for hemolysis and is usually caused by malpositioning or migration of the device cannula; kinking or pannus on the outflow cannula and graft thrombus formation within the pump, on the inlet and outlet stators (but not the rotor); or aortic insufficiency [46]. Patients can also have anemia without bleeding, which is accompanied by increased bilirubin, LDH, and plasma free hemoglobin levels and an undetectable haptoglobin level.

The role of imaging in determining the cause of hemolysis is unclear. TEE may be beneficial for assessing cannula velocities. Computed tomographic imaging may be helpful for diagnosing a mechanical inflow cannula obstruction.

Treating hemolysis can be challenging. The device speed may need to be adjusted downward. Glycoprotein IIb/IIIa inhibitors have also been used [47]. When this type of antiplatelet therapy is not tolerated, pentoxifylline has been used to improve blood flow. Plasmapheresis has also been undertaken with some success. The definitive therapy is device exchange if no reversible cause of hemolysis is identified.

### Chest pain

Chest pain in patients with LVADs is a complex issue but is most commonly due to noncardiac causes [12]. The true incidence of ischemic chest pain during LVAD support remains unknown.

Cardiac causes of chest pain include acute MI, unstable angina, pericarditis, myocarditis, and aortic dissection. Management of a new MI has been described above. Aortic dissection has been reported in patients undergoing LVAD support, especially those with the pulsatile Novacor device (WorldHeart Corp.) [48] and the HeartMate XVE (Thoratec Corp) device in the postoperative phase [49]. However, these devices are now essentially obsolete. TEE is used to identify aortic dissection. Sometimes the competition of the retrograde pump flow and the anterograde native blood flow may give an impression of an aortic dissection on CT angiography and TEE, which can be resolved by lowering the pump speed [50, 51].

Other noncardiac causes of chest pain in a patient with an LVAD are pulmonary embolus, which is rare [52], pneumothorax, esophageal tear, cholecystitis, pancreatitis, pneumonia and pleuritis, pneumomediastinum, esophageal spasm, gastroesophageal reflux disease, peptic ulcer disease, biliary colic, costochondritis, rib injury or fracture, arthritis, spinal root compression, or postherpetic neuralgia.

### Syncope

Syncope can be cardiac in origin, caused by hypotension (all causes of low CO as above), arrhythmias, or aortic valve thrombosis [53], or it can be noncardiac in origin. Trauma, GI bleeding, rupture of an aortic aneurysm, a ruptured ovarian cyst, or a ruptured spleen decrease effective blood volume and, thus, can lead to syncope. Other causes are hypoxemia, pulmonary embolism, subarachnoid hemorrhage, vertebrobasilar transient ischemic attack, subclavian steal syndrome, and neurocardiogenic syncope. Pregnancy has been reported in patients with LVADs; therefore, for young women, ectopic pregnancy should be ruled out as a cause of syncope [54]. Orthostatic left atrial and LV chamber collapse have been reported after postural change [55]. Fludrocortisone and midodrine have been used to treat syncope. Ruling out seizures is necessary.

A variety of means can be used to determine the cause of the syncope. A detailed physical examination and device interrogation should be performed. Laboratory tests to consider include a complete blood count, an electrolyte and metabolic panel, serum glucose evaluation, coagulation panel, arterial blood gas values, lactate levels, urinalysis, urine drug screen, and blood and urine cultures (if syncope and hypotension with sepsis or septic shock are differential diagnoses). Electrocardiography and AICD interrogation are valuable. Echocardiography can be used to assess the possibility of tamponade, RV dysfunction, aortic valve opening, and thrombosis. CT of the head should be done with a low threshold as patients are usually taking anticoagulants. A CT scan can also be used to rule out pulmonary embolism and aortic dissection. All patients with syncope should be considered for hospital admission.

### Gastrointestinal issues

The LVAD-related causes of GI symptoms are GI bleeding, hemolysis, hepatitis, pancreatitis, hepatic dysfunction, mesenteric ischemia or infarction, MI, pneumonia, pulmonary embolism, uremia, electrolyte abnormalities, neurologic causes like hypoperfusion and intracranial bleeding, low CO states, sepsis, and prescribed drugs. Gastroenteritis, diverticulitis, appendicitis, viscus perforation, small bowel obstruction, biliary tract disease, ureteral colic, urinary tract infection, diabetic ketoacidosis, herpes zoster, muscle spasm, and pregnancy are non-LVAD-related causes of nonspecific GI symptoms.

#### Dysphagia

Intravascular hemolysis has been linked to smooth muscle dystonia, which results in dysphagia and abdominal pain in patients with LVADs. An excessive level of plasma free hemoglobin can result in dystonia of the GI system [56, 57] and could indicate ongoing hemolysis. Symptoms should subside with such therapies as intravenous tissue plasminogen activator, pump exchange, or both.

#### GI bleeding

The incidence of GI bleeding in LVAD patients has been reported to be approximately 22 to 40 % [58]. The cause of the bleeding is thought to be due to the altered blood-flow patterns of continuous flow and also to acquired von Willebrand factor deficiency [59]. The minimal opening of the aortic valve creates flow patterns similar to that in patients with aortic stenosis (called Heyde syndrome), which leads to abnormal pulse waves, intestinal hypoperfusion, and distension of the submucosal venous plexuses of the GI tract. This, in turn, leads to angiodysplasia, arteriovenous malformations, and bleeding—eventually with minimal shear stress. Another possible mechanism of GI bleeding in these patients is acquired von Willebrand disease type 2A, which is caused by a deficiency of high molecular weight multimers of von Willebrand factor. This deficiency can occur in patients with continuous-flow LVADs secondary to shear stress on red blood cells [60, 61]. In addition, the contact between blood and the foreign surfaces of the LVADs may alter the rheology of blood flow, leading to a state of compensated coagulopathy. This process does not continue after cardiac transplantation. Gastric antral vascular ectasia (GAVE) syndrome is another rare cause of GI bleeding in patients with an LVAD. On endoscopy, the typical pathognomonic patterns seen for GAVE are red spots organized in stripes radiating from the pylorus (often described as *watermelon stomach* and usually seen in patients without cirrhosis) or arranged in a diffusely punctuated manner (the so-called honeycomb stomach, which is more common in cirrhosis) [62, 63].

In a few cases, octreotide, a long-acting somatostatin analog that decreases splanchnic arterial and portal blood flow, has been used to treat GI bleeding [64, 65]. The patients treated with octreotide for chronically bleeding arteriovenous malformations had fewer hospital admissions and administered blood units and had mean hemoglobin values that were higher than would be typical for patients with this condition.

**Fig. 5:**
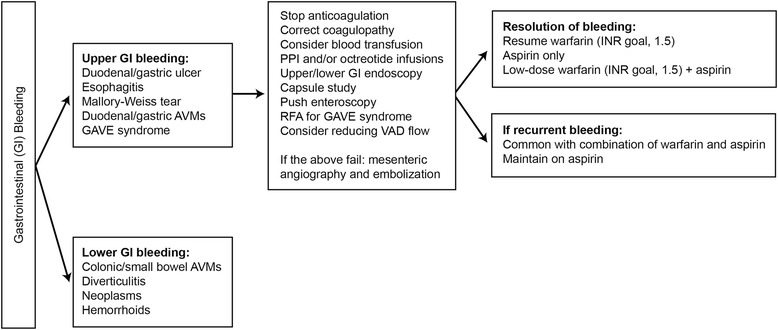
Gastrointestinal bleeding in patients with mechanical cardiac assist devices. *AVM* arteriovenous malformation, *GAVE* gastric antral vascular ectasia, *GI* gastrointestinal, *INR* international normalized ratio, *PPI* proton pump inhibitor, *RFA* radiofrequency ablation, *VAD* ventricular assist device

An algorithm for diagnostic and therapeutic approaches to patients with GI bleeding is summarized in Fig. 5.

### Acute kidney injury

#### Oliguria and anuria

Patients may present with a new onset of acute kidney injury (AKI). They may report oliguria, abdominal pain, fatigue, nausea, vomiting, dysuria, hematuria, and lower extremity edema. The incidence of AKI after LVAD implantation has been reported to range from 7 to 56 % [66], with the large variation in range likely due to differing definitions for AKI, the extent of heart failure, and the severity of preexisting kidney disease [66]. Most patients have improved kidney function after device implantation. The mortality rate is high for those patients who develop AKI after LVAD implantation.

**Fig. 6:**
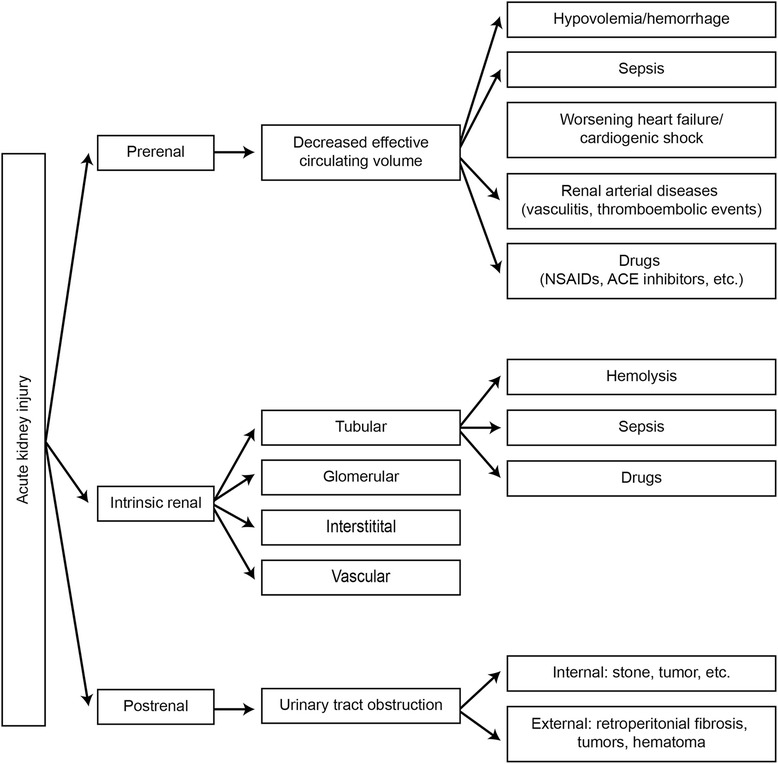
Diagnostic and management approach to patients with acute kidney injuries. *ACEI* angiotensin-converting enzyme inhibitors, *ARB* angiotensin receptor blockers, *NSAID* nonsteroidal anti-inflammatory drug

Figure 6 shows the possible causes of AKI in patients who have an LVAD. Poor forward flow due to device malfunction or LV dysfunction, as well as pigment nephropathy from hemolysis, is important in the process of differential diagnosis. The diagnostic approach to AKI is also summarized in Fig. 6. Patients who do not improve after optimizing hemodynamic parameters and volume status may need renal replacement therapy. Criteria for renal replacement therapy are the same for patients with or without LVADs, i.e., acidosis, electrolyte imbalance (hyperkalemia, hyperphosphatemia), fluid overload, and uremic encephalopathy and pericarditis. Emergency dialysis will require temporary catheter placement. For long-term hemodialysis, arteriovenous graft placement is preferred to avoid the high risk of infection associated with central venous catheters. Although there are no studies that describe maturation of arteriovenous fistulas in patients with LVADs, theoretical considerations of nonpulsatile flow have led authors to believe that an arteriovenous fistula should not be considered a vascular access option [66]. Peritoneal dialysis can also be used with the newer VADs, which are implanted in the precordial space or preperitoneal space behind the rectus sheath. Doppler audible ultrasonography should be used to assess arteriovenous-graft patency.

#### Hematuria

Hematuria in a patient with an LVAD could be either VAD-related or non-VAD-related. Intravascular hemolysis (hemoglobinuria), rhabdomyolysis, anticoagulation, medication-induced thrombotic thrombocytopenic purpura, and bacterial endocarditis are VAD-related causes of hematuria. Drugs, urinary tract infection, nephrolithiasis, benign prostatic hyperplasia, prostate, bladder, and renal malignancies, and autoimmune diseases are non-VAD-related causes of hematuria.

The following laboratory and imaging tests should be ordered to determine the cause of the hematuria: urinalysis; urine culture; plasma free hemoglobin, LDH, and haptoglobin levels; coagulation panel, ADAMTS13 activity and inhibitor profile (if thrombotic thrombocytopenic purpura is a consideration); renal function panel; urine eosinophil level; ultrasonography of the kidneys or CT imaging; and cystoscopy. A urology consultation may also be considered.

### Infection

Continuous-flow LVADs have lower overall infection rates compared with pulsatile devices but the rates still range from 30 to 50 % in patients with implanted devices [67, 68]. Patients who undergo LVAD implantation for destination therapy are more likely to develop infections than patients who had LVADs implanted as a bridge to transplantation because destination therapy patients tend to be sicker and have longer durations of LVAD support.

The diagnosis of sepsis in patients with LVADs is the same as that for other patient populations. Important steps in the management of sepsis include blood cultures, antibiotics, measuring lactate levels to assess for a state of vasodilatory shock and reduced tissue perfusion, and intravenous fluids, as deemed necessary based on the hemodynamic assessment. Patients may be hypotensive and have vasodilatation, with high VAD flow rates. Therefore, vasopressor agents may be needed early in the management course. Central access and measurement of central venous pressure for assessment of fluid responsiveness may help in guiding therapy. Inotropic agents may be needed if septic cardiomyopathy leads to RV dysfunction. VADs are related directly or indirectly to a variety of infections, including the VAD-specific infections (pump pocket, cannula, driveline) and VAD-related infections (endocarditis and pericarditis). VAD patients are also susceptible to mediastinitis and pneumonia, central line infections, e.g., a PICC line (peripherally inserted central catheter), or other non-VAD-related infections, such as urinary tract infections. Meticulous head-to-toe assessment is necessary to consider all possible sources of infection in patients who meet sepsis criteria [68, 69].

#### Driveline infections

Driveline infections occur in 17 to 30 % of patients with LVADs [68]. Cutaneous migration of bacteria and local trauma are causative factors and there may be cellulitis at the exit site. Ultrasonography and CT may be helpful for evaluating the condition of the driveline. Commonly seen organisms include species of *Staphylococcus*, *Enterococcus*, *Pseudomonas*, *Enterobacter*, and *Candida*. Oral or intravenous antibiotics should be administered according to the severity of the infection. Two weeks of therapy may suffice, although prolonged treatment may be necessary when the patient has bacteremia.

#### Pump-pocket infection

Pump-pocket infections occur in 2 to 10 % of patients with LVADs [68]. These infections may appear as an abscess beneath the skin or there may be purulent drainage and systemic signs (e.g., sepsis). Ultrasonography and computed tomography can be used in the diagnosis of pump-pocket infections. *Staphylococcus* species is a common cause; Gram-negative bacteria and *Candida* species have also been reported. Management includes drainage and debridement of the device pocket and empiric, broad-spectrum antibiotics. Long-term antimicrobial suppressive therapy may be indicated. Wrapping the omentum has been described to help prevent pocket infections [68].

#### Cannula or endocarditis (device) infection

Although rare (approximately 0.6 %) [68], infection occurring in the device or a cannula is associated with a high mortality. Diagnosis is usually presumptive when other sources of infection cannot be found and patients generally present with septic shock. Device removal is necessary and urgent transplantation is the preferred treatment for bridge-to-transplant patients. Chronic antibiotics may be necessary for destination-therapy patients.

#### Bloodstream infection

Bloodstream infections are common, with a reported rate of 20 to 27 % [68]. Patients with bloodstream infections can have fever, leukocytosis, septic shock, and septic embolization, which may emanate from the central catheter or be LVAD-related. If blood cultures from a peripheral and central catheter grow the same organism less than 2 h apart, this is usually a sign of LVAD-related bacteremia. More than 2 h between the two cultures could indicate a catheter-related infection. Empiric antibiotics should be given and the central catheter should be removed. If the bacteremia continues, the device should be exchanged or a heart transplant done, although both of these procedures are associated with a low survival rate [68].

Other possible sources of infection are pneumonia, urinary tract infections, sinusitis, cholecystitis, wound infections, and cellulitis.

### Neurologic symptoms

The initial assessment of an LVAD patient with altered mental status or new-onset focal or global neurologic deficits should be similar to that done for any patient with this condition and should include a primary survey and an assessment of the device. The LVAD coordinator and the cardiac surgery team should be contacted. Causes of altered mental status and confusion could be related to low-flow states and hypotension, stroke, seizure, infections, medications, electrolyte or metabolic abnormalities, hypoxia, hypercarbia or trauma, and thromboembolic causes. In addition to routine workup for the above, a complete blood count, a hemolysis panel (LDH/haptoglobin, bilirubin, and plasma free hemoglobin levels), and a coagulation panel should be ordered.

With the introduction of continuous-flow devices, the incidence of stroke in patients with LVADs has decreased substantially; however, patients remain at a high risk for stroke [70]. Of the neurologic events associated with LVADs, ischemic stroke is most prevalent but hemorrhagic stroke has a higher mortality rate [71]. A study comparing the use of pulsatile-flow LVADs and continuous-flow LVADs showed the rate of hemorrhagic stroke to be 11 % in the continuous-flow group and 8 % in the pulsatile-flow group [72]. Risk factors for ischemic stroke in patients with LVADs include diabetes, having had a previous stroke, aortic cross-clamping with cardioplegic arrest during the LVAD implantation procedure [73], and systemic infection [74].

Neurologic events in patients with LVADs are generally related to several factors. First, patients with LVADs take anticoagulation and antiplatelet medications chronically to prevent thrombosis in the device. No universally accepted protocol exists for anticoagulation and antiplatelet therapy after LVAD insertion, although most regimens include postoperative heparin with a transition to warfarin, aspirin, and potentially dipyridamole or clopidogrel [75]. The need for anticoagulation predisposes these patients to intracranial hemorrhage and hemorrhagic stroke, as well as to other sequelae related to bleeding [75]. Second, LVAD patients can develop acquired von Willebrand syndrome, as described above, which seems to be related to the shear stress the device exerts and to impaired hemostasis of the vascular endothelium. Third, infection is also associated with stroke. Fourth, device thrombosis from a source outside the LVAD or formed within the device is a well-known cause of embolic stroke in patients. Of all strokes that occurred, 58 % were found to occur in the right hemisphere, 28 % in the left hemisphere, and 6.5 % in both hemispheres. In addition, about 6.5 % of strokes are vertebrobasilar in origin [76]. The predilection for right hemispheric stroke was explained by the anatomic alignment directing thrombotic material toward the brachiocephalic trunk [76].

Protocols exist for the acute management of stroke in LVAD patients [77] and begin with contacting the neurology team. Anticoagulation therapy should be considered for patients with ischemic stroke and coagulopathy needs to be reversed for patients with hemorrhagic stroke. The most common approaches for managing stroke follow.

#### Ischemic stroke

CT and CT angiography should be used to determine the cause and extent of ischemic stroke. The risks and benefits of systemic thrombolytic agents need to be discussed among the cardiology, cardiac surgery, neurology, and critical care teams. If there is a large-vessel occlusion, an endovascular procedure can be considered.

#### Hemorrhagic stroke

If a patient has been taking warfarin, prothrombin complex concentrate or fresh frozen plasma should be administered. Vitamin K can also be given. Desmopressin and a platelet transfusion should be considered for those patients taking antiplatelet drugs. If systemic thrombolytic therapy has been administered and an intracranial hemorrhage is noted subsequently, cryoprecipitate and antifibrinolytic agents, such as ε-aminocaproic acid or tranexamic acid, should be considered.

#### Headache

Headaches have several possible causes. Central nervous system causes include intracranial hemorrhage and reversible posterior leukoencephalopathy syndrome [78]. Cardiopulmonary causes include hypoxic and hypercarbic respiratory failure, low CO, and hypertension. Although primary central nervous system infections are rare, secondary seeding from bloodstream infections and endocarditis may occur and should be investigated. Other causes of headache unrelated to the LVAD include medications, migraine headache, toothache, eye pain, sinusitis, and musculoskeletal pain.

### Back pain

Causes of back pain that are VAD-related include epidural hematoma, abscess, retroperitoneal bleeding, aortic dissection, and pulmonary embolism. Non-VAD specific reasons for back pain include cauda equina, spinal fracture, disk herniation, osteoarthritis, osteomyelitis, spinal stenosis, and muscle sprain. Other causes include cholecystitis, pancreatitis, peptic ulcer diseases, nephrolithiasis, ovarian torsion, pulmonary embolism, pneumonia, and tumors.

### Brain death and organ donation

Neurologic events during LVAD support are associated with significant morbidity and mortality rates and can cause brainstem death. A patient with diagnosed brain death who is supported by a VAD and has preserved end-organ function can and should be considered for donating organs [79], including the liver, kidneys, and lungs. In some cases, organ donation has also occurred after cardiac death.

### Cardiac arrest

Cardiac arrest may occur in patients whose circulation is supported by an LVAD. In these cases, a stepwise approach to management should be used. The VAD coordinator should be contacted immediately. All of the VAD equipment should be assessed to verify that critical connections are intact. The driveline and power supply should be checked and reconnected if disconnected. Alarms should be assessed. If there is a VAD hum, it should be assessed with auscultation. Doppler ultrasonography should be used to evaluate the patient’s blood pressure. If the pump is off, the backup controller should be switched on or, alternatively, the power sources should be switched. Advanced cardiac life support should be continued without chest compression. (Although chest compression was not reported as harmful in a case series [80], the current recommendation is for no chest compression.) The major risk associated with chest compression during cardiopulmonary resuscitation is dislodgement of the device or its outflow cannula, which is located directly beneath the sternum. A potential alternative to chest compression is abdominal compression 1 to 2 inches left of midline, which has been described [81] but is not currently recommended. Other than that noted above, management is done per advanced cardiac life support guidelines and includes the use of epinephrine and cardioversion.

### Orthopedic injuries

Fractures may require operative fixation. Because of the possibility of fat embolism during the intraoperative and postoperative periods and subsequent RV failure, patients with LVADs should be monitored closely. Transcranial Doppler ultrasonography can be used to help monitor cerebral perfusion.

### Psychiatric and pyschological issues

Patients with LVADs may have psychiatric and psychological issues [82], such as depression, anxiety, paranoid ideation, sleep disturbances, somatization, psychosis, and obsessive-compulsive behavior. Some of these issues may have existed before device implantation because of the stress and effects of chronic heart failure and reduced perfusion to the brain. Insertion of an LVAD is reported to improve neurocognitive symptoms, including substantial improvements in memory in advanced heart failure patients [83]. However, acute psychiatric or psychological presentations in patients with LVADs should be investigated for organic causes like thrombosis, new stroke, and seizures. Monitoring a patient’s cognitive status is necessary to maximize the benefits of therapy and enhance quality of life.

### Noncardiac surgery

Noncardiac surgery can be accomplished safely while patients are being supported with a mechanical circulatory assist device. In a case series, 20 patients underwent 25 noncardiac, nonlaparoscopic surgical operations, including repair of inguinal hernia, cholecystectomy, small- and large-bowel surgery, and other gynecologic procedures; there were few complications and no deaths [84]. Bleeding requiring blood transfusion and operative reexploration was the most serious complication. However, it occurred less often in patients who discontinued taking warfarin before the operation. Other reports have described the relative safety of discontinuing warfarin in patients with a contraindication to anticoagulation and an implanted LVAD [85, 86]. Therefore, warfarin can be discontinued for 5 days before a major operative procedure to reduce the risk of postoperative bleeding [84]. In addition, heparin can be used as a bridge for those patients who need anticoagulation therapy because of atrial fibrillation or LV thrombus [84]. Fresh frozen plasma and vitamin K can be used to rapidly reverse the anticoagulation therapy when an emergency operation is needed for a patient with an implanted device; this has been done without an increased incidence of device thrombosis.

### Trauma

When a patient with an LVAD presents with trauma, device malfunction needs to be assessed immediately (as described above), including by radiography and CT, if necessary. Advanced Trauma Life Support guidelines (American College of Surgeons) should be followed to rule out traumatic subarachnoid hemorrhage or subdural hematoma, pneumothorax, hemothorax, pulmonary injuries, intraabdominal organ injuries, and spinal cord trauma. The VAD coordinator and CT surgery team should be contacted. Trauma-related failure of a continuous-flow LVAD has been reported [87]. Damage to the cables and displacement of the pump from its original position happened after patients fell or were hit in the chest.

### Biventricular assist devices

The incidence of RV failure requiring an RVAD has decreased with the transition to continuous-flow devices [88–90]. Only 4 % of patients in the HeartMate II BTT trial required RVAD support [71]. In addition, risk-assessment tools have been developed to help determine which patients might need BiVAD support [91, 92]. Biventricular support is more complicated to manage and, to avoid pulmonary edema, LV output must always be greater than RV output [93]. Native RV and LV function may also recover at different rates.

Two devices are used for BiVAD support: the Thoratec PVAD/IVAD (Thoratec Corp.) and the HeartWare. The PVAD/IVAD system provides pulsatile flow and the HeartWare (as described previously) provides continuous flow [88, 89]. The PVAD system has three main components: a blood pump, cannulas, and a pneumatic driver (Dual Drive Console or TLC-II Portable VAD Driver). The Thoratec IVAD is an implantable version of the Thoratec PVAD. Simultaneous LV and RV support using dual HeartWare pumps is common but the incidence of right-sided HVAD pump thrombosis is high. To maintain lower flows in the pulmonary system, apart from controlling speeds, a pinched valve or smaller-diameter outflow graft [88, 90] have been reported for use on the right side. The mortality rate for patients undergoing BiVAD support increases as the length of time on biventricular support increases. The HeartMate 3, currently undergoing clinical trials (MOMENTUM 3), may also be used eventually for BiVAD support.

A few reports in the literature describe experiences in discharging a small number of BiVAD patients home to await transplantation [62, 94, 95]. The three leading causes of readmissions in one study were GI bleeding (23 %), pulsatile ventricular assist device (PVAD) thrombosis (17 %), and infections at the site of VAD placement (15 %), which were consistent with complication rates from other studies after VAD implantation [95]. Managing treatment with a BiVAD is similar to managing treatment with an LVAD, although the RV and LV output can be pulsatile or continuous, depending on the device used.

### Total artificial heart

The TAH is designed to replace the cardiac structures completely and to provide long-term support as a BTT. A TAH is considered for those patients in whom biventricular support is required. The SynCardia TAH (formerly called *CardioWest*; SynCardia Systems, Inc.) is a biventricular pneumatic pulsatile pump that replaces the native ventricle and all four heart valves [96]. The SynCardia TAH has been approved by the US Food and Drug Administration (FDA) as a BTT and received a recent approval from the FDA for a clinical study of effectiveness for destination therapy. The two pumps are placed in the orthotopic position after polyurethane inflow connectors are sutured to the left and right atrial cuffs of the recipient [97]. Dacron outflow grafts connect the TAH to the pulmonary artery and aorta. A percutaneous driveline connects to the system controller (either the pneumatic Freedom portable driver [SynCardia Systems, Inc.] or the nonportable hospital driver [often called *Big Blue*]). The device will pump up to 9.5 L/min through both ventricles; blood is pumped by the action of internal, pneumatically driven diaphragms. The design of the TAH (partial fill and full eject) allows for a more physiologic response, i.e., the TAH accepts and pumps blood on the basis of the patient’s need—either at rest or exercise—without requiring an adjustment for heart rate [97]. Thus, the device settings are patient-specific, based on the afterload pressures of the aorta and pulmonary artery [97, 98].

The adverse events that occur with TAH support are similar to those in patients with LVADs. Respiratory failure, infections, bleeding, anemia, hemolysis, neurologic events, hepatic dysfunction, peripheral thromboembolism, and renal failure may occur. It is important to remember that patients with an implanted TAH *will* have a pulse, whereas patients with an LVAD usually *will not* have a pulse. Patients with a TAH *will not* have a cardiac rhythm, whereas patients with an LVAD *will* have a cardiac rhythm. For patients with a TAH, a pressure cuff is all that is needed for monitoring. CO and filling volume of the right- and left-sided pumps should be assessed frequently. Cardiopulmonary resuscitation is ineffective for TAH patients and inotropic agents will not change hemodynamic parameters.

Three types of alarms can occur: (1) a battery alarm, (2) a temperature alarm, and (3) a fault alarm [29]. Batteries should be replaced one at a time and reinstalled correctly. The temperature alarm indicates the internal temperature of the driver is too hot or the temperature of the onboard batteries is too hot. In this case, any objects that are blocking the filter cover, fan, or both should be removed. The filter cover should be inspected or the Freedom portable driver should be moved to a cooler area. A fault alarm can indicate a Valsalva maneuver, a kinked or disconnected driveline, a driver that is connected to external power without at least one correctly inserted onboard battery or a battery with less than 30 % charge, and malfunction of the driver. These possible problems should be considered to address the alarm. A backup Freedom portable driver should be available in the case of driver malfunction.

## Conclusions

Current, continuous-flow mechanical circulatory assist devices are smaller, more durable, safer, and more effective for treating patients with severe heart failure than their pulsatile-flow predecessors. With the FDA’s market approval of the HeartMate II, more patients are having these devices implanted, and additional devices are currently undergoing clinical trials. Thus, health care providers in EDs and ICUs need to have a good knowledge of the symptoms and signs associated with complications of these devices and be able to make the differential diagnosis and begin the management steps required for a successful outcome for these patients. A multidisciplinary team-based approach is the first essential step to improved patient outcomes.

## Abbreviations

AICD: automated implantable cardioverter defibrillator
AKI: acute kidney injury
BiVAD: biventricular assist device
BTT: bridge to transplant
CO: cardiac output
CT: computed tomography
DT: destination therapy
ED: emergency department
FDA: US Food and Drug Administration
Fio_2_: fraction of inspired oxygen
GAVE: gastric antral vascular ectasia syndrome
GI: gastrointestinal
ICU: intensive care unit
LDH: lactase dehydrogenase
LV: left ventricle/left ventricular
LVAD: left ventricular assist device
MAP: mean arterial pressure
MI: myocardial infarction
PEEP: positive end-expiratory pressure
PI: pulsatility index
PVAD: pulsatile ventricular assist device
RPM: revolutions per minute
RV: right ventricle/right ventricular
RVAD: right ventricular assist device
TAH: total artificial heart
TEE: transesophageal echocardiography
VAD: ventricular assist device
